# A Comparative Study on Outcomes and Quality of Life Changes Following Ventral Transabdominal Preperitoneal (Ventral-TAPP) and Laparoscopic Intraperitoneal Onlay Mesh (IPOM)-Plus Repair for Ventral Hernia

**DOI:** 10.7759/cureus.42222

**Published:** 2023-07-20

**Authors:** Kumar Kaushik, Vivek Srivastava, Anumanchi Datta Sai Subramanyam, Ritwik Kishore, Arvind Pratap, Mumtaz A Ansari

**Affiliations:** 1 Department of General Surgery, Institute of Medical Sciences, Banaras Hindu University, Varanasi, IND

**Keywords:** ventral-tapp, eurahs-qol, ppom, ipom-plus, laparoscopic ventral hernia repair (lvhr), incisional hernia

## Abstract

Background

Ventral transabdominal preperitoneal (ventral-TAPP) repair and intraperitoneal onlay mesh (IPOM) plus repair are two options among the available techniques of laparoscopic ventral hernia repair (LVHR). We conducted a comparative study to evaluate the clinical and quality of life (QoL)-related outcomes between ventral-TAPP and IPOM-plus repair. It was hypothesized that both procedures offered similar outcomes and QoL.

Materials and methods

The study included 32 consecutive patients undergoing LVHR, divided equally between ventral-TAPP and IPOM-plus groups. In the ventral-TAPP procedure, a peritoneal flap was created around the defect, followed by defect approximation and polypropylene mesh placement in the pre-peritoneal pocket. For the IPOM-plus procedure, the defect was closed and a composite (dual-side) mesh was placed around the defect. The minimum overlap beyond the original defect margin in both groups was 5 cm. Data regarding pre-operative parameters and postoperative outcomes, including pain and QoL at one week, one month, and three months, were recorded. A p-value of less than or equal to 0.05 was considered to be statistically significant.

Results

While the mean duration of surgery was longer, the cost of treatment was lower in group 1 (ventral-TAPP) with a p-value of <0.05 for both parameters. The length of hospital stay was significantly shorter in group 1 (ventral-TAPP), while the return to normal activity was similar in both groups. The visual analog scale (VAS) score for overall pain perception and the European registry for abdominal wall hernias (EuraHS; hernia-related QoL) score for ‘Pain at Site’ and ‘Restriction of Activity’ domains were significantly higher in group 2 (IPOM-plus) at one week.

Conclusion

Although the ventral-TAPP procedure requires more time and expertise to perform, the EuraHS QoL assessment at one week was better in group 1 (ventral-TAPP). Ventral-TAPP group scored better in terms of length of hospital stay and cost-effectiveness as well.

## Introduction

In our healthcare system, ventral hernia and their recurrences pose a significant economic burden [1]. The incidence rate of ventral incisional hernias is estimated to be around 2-20% [2]. Surgery for ventral abdominal wall hernias, which is a frequent intervention, has traditionally been managed using open mesh hernioplasty techniques [3]. Open mesh repair involves inlay, onlay, sublay, and underlay mesh placement.

Laparoscopic ventral hernia repair (LVHR) has been gaining acceptance since its introduction in 1993 due to its superior outcomes compared to open ventral hernia repair [4,5]. Nevertheless, there remains a significant amount of controversy surrounding the optimal approach to LVHR. Intraperitoneal onlay mesh (IPOM) repair is a technique where a hernia defect is bridged from the peritoneal side using a mesh. This minimally invasive procedure requires smaller surgical incisions, which reduces postoperative pain, improves cosmesis, and avoids extensive tissue dissection [6]. IPOM-plus repair, on the other hand, is a variation of this technique where the hernia orifice is closed with a suture before the mesh is placed [7].

With the rise in cases of mesh-induced visceral complications, concerns have grown over the plane of mesh placement. Consequently, laparoscopic techniques have been devised to place the mesh extraperitoneally [8]. The ventral transabdominal preperitoneal (ventral-TAPP) is one such approach. This method minimizes damage to the architecture and structures of the abdominal wall. Placing the mesh in the preperitoneal plane avoids its contact with the bowel and other intra-abdominal contents. Compared to IPOM-plus, ventral-TAPP involves a longer operative time and requires expertise in dissection to create the peritoneal flap. The surgeon's orientation during the creation of the peritoneal flap is a crucial aspect [9]. The ventral-TAPP approach has also been referred to as the laparoscopic preperitoneal onlay mesh method [10].

In the current literature, there is published data on fascial defect closure techniques and mesh handling, but the question of the standard procedure remains unresolved. In this study, we compared the two types of LVHR procedures, ventral-TAPP and IPOM-plus, in relation to outcomes and hernia-related quality of life (QoL).

## Materials and methods

The study was conducted in the general surgery department at a tertiary care center in India from July 2020 to June 2022, following approval from the institute's ethical committee. Patients presenting with primary ventral or incisional hernias underwent basic serum tests and abdominal ultrasonography or contrast-enhanced CT scan. Those with a confirmed hernia defect size of ≤10 cm who were undergoing laparoscopic repair for ventral hernia were enrolled after providing informed written consent. Patients with complicated, recurrent (i.e., more than two times) hernias, and serious comorbidities, as well as elderly and pediatric patients, were excluded from the study. The study includes 32 consecutive ventral hernia patients suitable for LVHR. These patients were alternatively assigned to two groups: group 1 for ventral-TAPP and group 2 for IPOM-plus.

**Figure 1:**
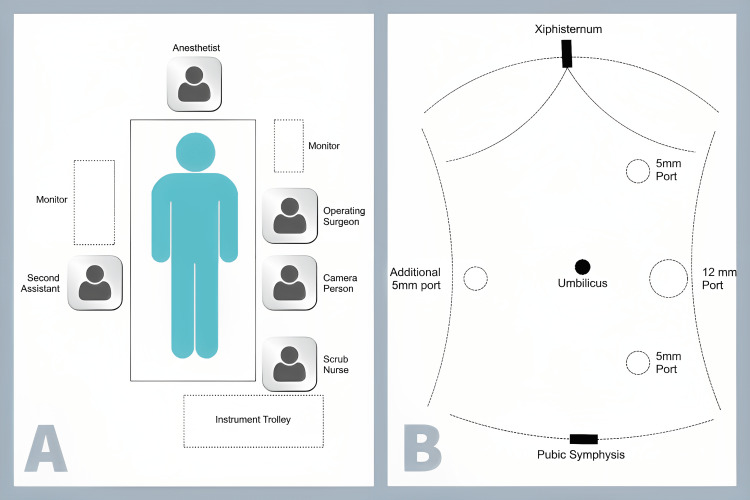
Schematic diagram of (A) operation theater layout and (B) port positions

Before the surgical procedure, antibiotics were administered and the skin was prepared. The patient was placed in a supine position and put under general anesthesia with endotracheal intubation. Foley catheterization was performed to decompress the urinary bladder, and both arms were tucked into the trunk without any elevation from the horizontal plane. For non-midline hernias, the operating table was tilted with slight ipsilateral elevation. A Veress needle was used to create a pneumoperitoneum (10 mm to 12 mmHg) from Palmer's point access. The first trocar was inserted at Palmer’s point, and a 5 mm 30° optic was used to perform diagnostic laparoscopy. The hernial defect was marked along with the mesh, ensuring a 5 cm overlap all around the defect. For ventral-TAPP, an additional marking of the peritoneal pocket size (2 cm around the mesh border) and peritoneal incision line was made. Two or three working ports were used, depending on the area and position of the defect, with the third working port in the contralateral (right) flank as shown in Figure 1. The middle left lateral port was made 12 mm in size for mesh delivery. This procedure was followed in most cases; however, the port position was altered in a few operations to ensure proper ergonomics and safe surgery.

For both the ventral-TAPP and IPOM-plus procedures, the adhesiolysis was primarily carried out using blunt and sharp scissor dissection, and the contents of the hernia were reduced. A reassessment of the hernia defect and the required mesh size was then performed.

In the ventral-TAPP procedure, a peritoneal flap was created around the hernia orifice at this stage. This required careful dissection with cautery scissors using short bursts. The hernia orifice was then closed using a non-absorbable barbed polybutester suture. A polypropylene mesh was placed inside the peritoneal pocket and fixed with transfascial sutures and a few tackers. The peritoneum was then sealed with a running absorbable polyglactin suture.

For the IPOM-plus procedure, the area for mesh placement was cleared of fatty tissue, omentum, and ligaments using a vessel sealing energy device. The defect was then closed with the same non-absorbable barbed suture. An appropriately sized composite mesh (polyester mesh with a collagen barrier) was then hydrated, rolled in, aligned, and tacked in place.

During follow-up, patients were assessed for complications, the visual analog scale (VAS) score for pain, and the European registry for abdominal wall hernias (EuraHS; hernia-related QoL) score at one week, one month, and three months, respectively. Complications included subcutaneous hematoma, deep vein thrombosis, seroma formation, wound infection, mesh bulging, recurrence, intestinal obstruction, sinus formation, and enterocutaneous fistulation. The EuraHS QoL assessment is a hernia-specific questionnaire with nine questions divided into three domains: pain (three questions), restriction of activity (four questions), and cosmetic discomfort (two questions). The cumulative minimum score (representing the best QoL) is 0, and the cumulative maximum score (representing the worst QoL) is 90 [11]. We analyzed the results by averaging the scores documented for questions in their respective domains and calculating the mean value.

Data was collected using a pre-structured proforma during hospital stays and follow-ups. This included demographic information, clinical findings, and perioperative variables such as operative time, hospital stay duration, and treatment costs. The time taken by the patient to resume normal activity was recorded as well. Statistical analysis was performed using SPSS Statistics version 25.0 (IBM Corp. Released 2017. IBM SPSS Statistics for Windows, Version 25.0. Armonk, NY: IBM Corp.), with a p-value of ≤ 0.05 considered statistically significant.

## Results

In our study, 32 patients underwent LVHR, with 16 undergoing the ventral-TAPP procedure (Group 1) and 16 undergoing the IPOM-plus procedure (Group 2). The mean age of patients was 43.8 years in Group 1 and 45.3 years in Group 2, a difference that was not statistically significant. Group 1 consisted of 56.3 percent male patients, while Group 2 consisted of 43.8 percent male patients. The predominance of males in Group 1 and females in Group 2 was statistically significant, with a p-value of 0.035 (Table 1).

**Table 1:** Comparison of demographic and clinical parameters, risk factors, and comorbidities between both groups (vTAPP and IPOM-plus) vTAPP: ventral transabdominal preperitoneal repair, IPOM-plus: intraperitoneal onlay mesh plus repair, LUTS: lower urinary tract symptoms, DM: diabetes mellitus, HTN: hypertension, ASA: American Society of Anesthesiologists, #: one sample t-test statistical analysis, @: Chi-squared statistical analysis; *: p-value of ≤0.05 is significant

Parameters		Group 1 (vTAPP) (n=16)		Group 2 (IPOM-plus) (n=16)		Statistics	
		Frequency	Percentage	Frequency	Percentage	t-test/Chi-squared test	p-value
Age (yrs)		43.813	7.943	45.375	8.302	1.998^#^	0.059
Gender	Male	9	56.3	7	43.8	2.009^@^	0.035*
	Female	7	43.8	9	56.3		
Hernia type	Incisional	7	43.8	8	50.0	1.722^@^	0.069
	Umbilical	6	37.5	4	25.0		
	Epigastric	3	18.8	4	25.0		
	Lateral	0	0	0	0		
Number of defects	1	13	81.3	14	87.5	1.110^@^	0.056
	2	2	12.5	1	6.3		
	>2	1	6.3	1	6.3		
Fascial defect size of the hernia	<1 cm	7	43.8	8	50.0	2.290^@^	0.089
	1-2 cm	3	18.8	4	25.0		
	2-5 cm	5	31.3	3	18.8		
	5-10 cm	1	6.3	1	6.3		
Previous open hernia repair	No	13	81.3	12	75.0	1.556^@^	0.075
	Yes	3	18.8	4	25.0		
Constipation	No	15	93.8	12	75.0	1.916^@^	0.080
	Yes	1	6.3	4	25.0		
LUTS	No	13	81.3	13	81.3	-	-
	Yes	3	18.8	3	18.8		
Chronic cough	No	16	100	14	87.5	2.006^@^	0.058
	Yes	0	0	2	12.5		
Alcohol history	None	9	56.3	8	50.0	1.086^@^	0.056
	Occasional	5	31.3	6	37.5		
	Heavy	2	12.5	2	12.5		
Smoker	None	8	50.0	7	43.8	1.089^@^	0.078
	Occasional	6	37.5	8	50.0		
	Heavy	2	12.5	1	6.3		
DM	No	9	56.3	11	68.8	2.055^@^	0.081
	Yes	7	43.8	5	31.3		
HTN	No	13	81.3	8	50.0	1.446^@^	0.096
	Yes	3	18.8	8	50.0		
ASA grading	Grade 1	7	43.8	8	50.0	1.027^@^	0.079
	Grade 2	9	56.3	8	50.0		
	Grade 3	0	0	0	0		
	Grade 4	0	0	0	0		
	Grade 5	0	0	0	0		

Both groups were comparable in terms of hernia type, number of defects, and defect size (Table 1). The risk factors and comorbidities considered included constipation, lower urinary tract symptoms, chronic cough, alcohol intake, smoking habits, type 2 diabetes mellitus, hypertension, and a history of previous open hernia repair. No significant statistical difference was found between the two groups regarding these parameters. All patients were either American Society of Anesthesiologists ASA grade 1 or 2, making them comparable in this aspect as well (Table 1).

The mean duration of surgery was significantly longer for the ventral-TAPP procedure (171.3 minutes) compared to the IPOM-plus procedure (101.9 minutes), largely due to the additional step of peritoneal flap creation in the former. Moreover, the ventral-TAPP repair required a polypropylene mesh, which is less costly than the composite (dual-side) mesh used in the IPOM-plus repair. Therefore, the mean cost of treatment was Rs 20,060 in the ventral-TAPP group and Rs 63,600 in the IPOM-plus group. The p-value was less than 0.05 for both parameters mentioned above (Table 2). The mean length of hospital stay was 2.5 days for the ventral-TAPP procedure and 3.8 days for the IPOM-plus procedure, a difference that was statistically significant with a p-value of 0.03. The time taken to return to normal activities was comparable between the two groups (Table 2).

**Table 2:** Comparison of perioperative parameters between both the groups (vTAPP and IPOM-plus) vTAPP: ventral transabdominal preperitoneal repair, IPOM-plus: intraperitoneal onlay mesh plus repair, SD: standard deviation, *: p-value of ≤ 0.05 is significant

Parameters	Group 1 (vTAPP) (n=16)		Group 2 (IPOM-plus)		Statistics	
	Mean	SD	Mean	SD	t-test	p-value
Cost of treatment (thousand Indian Rupees)	20.06	5.01	63.60	15.50	2.227	0.002*
Duration of surgery (minutes)	171.37	11.28	101.93	13.49	1.998	0.004*
Length of hospital stay (days)	2.50	1.70	3.80	1.90	2.172	0.030*
Return to normal activities (days)	8.80	8.50	11.30	3.60	1.89	0.066

Patients were followed up at one week, one month, and three months postoperatively to assess complications, pain, and QoL. Among the complications considered, there were no incidences of deep vein thrombosis, sinus formation, mesh bulging, hernia recurrence, or enterocutaneous fistula formation. The occurrences of subcutaneous hematoma, seroma formation, wound infection, and intestinal obstruction are tabulated in Table 3. The IPOM-plus group reported a significantly higher rate of seroma formation than the ventral-TAPP group.

**Table 3:** Comparison of complications and VAS score for pain between the two groups (vTAPP and IPOM-plus) vTAPP: ventral transabdominal preperitoneal repair, IPOM-plus: intraperitoneal onlay mesh plus repair, VAS: visual analog scale, *: p-value of ≤0.05 is significant

Parameters		Group 1 (vTAPP) (n=16)		Group 2 (IPOM-plus)		Statistics	
		Frequency	Percentage	Frequency	Percentage	Chi-squared test	p-value
Subcutaneous hematoma_post op_1 week	No	14	87.5	15	93.8	1.809	0.094
	Yes	2	12.5	1	6.3		
Seroma formation_post op_1 week	No	16	100	14	87.5	1.443	0.004*
	Yes	0	0	2	12.5		
Seroma formation_post op_1 month	No	15	93.8	15	93.8	-	-
	Yes	1	6.3	1	6.3		
Seroma formation_post op_3 months	No	16	100	16	100	-	-
	Yes	0	0	0	0		
Wound infection_post op_1 week	No	16	100	16	100	-	-
	Yes	0	0	0	0		
Wound infection_post op_1 month	No	14	87.5	15	93.8	1.809	0.094
	Yes	2	12.5	1	6.3		
Intestinal obstruction_post op_1 week	No	16	100	16	100	-	--
	Yes	0	0	0	0		
Intestinal obstruction_post op_1 month	No	16	100	16	100	-	-
	Yes	0	0	0	0		
Intestinal obstruction_post op_3 months	No	16	100	15	93.8	2.667	0.078
	Yes	0	0	1	6.3		
VAS_post op_1 week	0	0	0	0	0	1.778	0.045*
	1-3	11	68.8	12	75.0		
	4-6	5	31.3	1	6.3		
	7-9	0	0	3	18.8		
	10	0	0	0	0		
VAS_post op_1 month	0	13	81.3	12	75.0	2.556	0.061
	1-3	1	6.3	3	18.8		
	4-6	2	12.5	1	6.3		
	7-9	0	0	0	0		
	10	0	0	0	0		
VAS_post op_3 months	0	15	93.8	15	93.8	-	-
	1-3	0	0	0	0		
	4-6	1	6.3	1	6.3		
	7-9	0	0	0	0		
	10	0	0	0	0		

The VAS was also used to estimate overall pain perception. At one week, the VAS was significantly lower in the ventral-TAPP group compared to the IPOM-plus group; however, there was no significant difference at the one-month or three-month follow-up (Table 3). An analysis of the EuraHS QoL score found higher mean scores for the "pain at site" and "restriction of activity" domains at the one-week follow-up in the IPOM-plus group, differences that were significant with p-values of 0.04 and 0.05, respectively. Scores in the "cosmetic discomfort" domain were comparable between both groups (Table 4).

**Table 4:** Comparison of EuraHS hernia-related QoL scores between the two groups (vTAPP and IPOM-plus) vTAPP: ventral transabdominal preperitoneal repair, IPOM-plus: intraperitoneal onlay mesh plus repair, EuraHS: European registry for abdominal wall hernias, *: p-value of ≤0.05 is significant

Parameters		Group 1 ( vTAPP ) (n=16)		Group 2 (IPOM-plus)		Statistics	
		Frequency	Percentage	Frequency	Percentage	t-test	p-value
EuraHS_pain at site_1 week		8.375	3.757	9.375	7.632	1.117	0.042*
EuraHS_pain at site_1 month		3.188	7.556	2.938	5.591	2.227	0.073
EuraHS_pain at site_3 months		1.000	4.000	1.063	4.250	1.099	0.0889
EuraHS_restriction of activity_1 week		5.813	9.005	6.563	12.094	1.718	0.051*
EuraHS_restriction of activity_1 month		4.625	10.620	4.000	7.677	1.516	0.074
EuraHS_restriction of activity_3 months		1.500	6.000	1.875	7.500	1.008	0.055
EuraHS_cosmetic discomfort_1 week		0.000	0.000	1.000	2.757	0.997	0.098
EuraHS_cosmetic discomfort_1 month		0.563	2.250	0.625	2.500	1.006	0.066
EuraHS_cosmetic discomfort_3 months		0.000	0.000	0.000	0.000	-	-

## Discussion

In 1993, LeBlanc and Booth pioneered the first documentation of laparoscopic LVHR [12]. However, the technique gained significant popularity after 2000, following the publication of a comprehensive multi-center series that emphasized the low complication rate and a hernia recurrence rate of only 3.4% associated with LVHR [4]. This seminal work spurred the broad adoption of LVHR as a minimally invasive alternative to the established Rives-Stoppa repair, hitherto regarded as the gold standard in abdominal wall reconstruction. The Rives-Stoppa repair marked a considerable advancement in this field, significantly reducing hernia recurrence through the use of retromuscular mesh with extensive overlap [13].

Basic laparoscopic hernia repair involves an intraperitoneal technique in which a mesh prosthesis is used to secure and cover the hernia defect. In this approach, the actual hernia defect is typically not closed but is instead patched with the mesh. Due to this characteristic, it becomes crucial to size the mesh appropriately to ensure adequate overlap (minimum 5 cm from the hernia margin) and to establish strong fixation for durability. This technique is referred to as IPOM, which is a bridging repair [14]. The IPOM-plus technique, on the other hand, reconstructs the abdominal fascia by first closing the defect and then adding the mesh support (augmentation repair).

Opting not to close the hernia defect in the IPOM technique and instead bridging the defect with mesh creates a functionally adynamic area. This raises the risk of bulging, seroma formation, and potential wound infection. Furthermore, closing the defect increases the overall surface area of the mesh-abdominal wall interface, promoting future tissue in-growth and strengthening the fixation. Therefore, technically, IPOM-plus is superior to IPOM [15]. However, the risk of visceral complications, caused by the mesh being in contact with the bowel, has led to the exploration of alternative laparoscopic approaches for ventral and incisional hernia repair.

Among the extraperitoneal planes, the retro-rectus space in the central abdomen is limited by its fascial compartment. To accommodate mesh placement across the right and left retro-rectus spaces, the linea alba must be disconnected, incised, and repaired. However, the unified retro-rectus compartment imposes limitations on the size of the mesh that can be utilized. To overcome this limitation and allow for the placement of larger meshes, laparoscopic transversus abdominis release is required, which involves accessing the preperitoneal plane [16].

The ventral-TAPP method for ventral hernia repair minimizes structural disruption to the abdominal wall architecture. The peritoneum itself has limited involvement in providing structural strength to the abdominal wall. In ventral-TAPP, the mesh placement leads to adhesion between the peritoneum and posterior fascia. In contrast, in retro-muscular techniques, the mesh results in adhesion between the muscle and fascial compartments, impeding the natural gliding of the muscles within that space. Therefore, compared to other methods such as component separation, ventral-TAPP may be considered to be a technique that causes the least structural disruption and minimizes instability of the abdominal wall [17].

Our study aimed to identify differences in outcomes and changes in QoL between the ventral-TAPP and IPOM-plus procedures. Our findings revealed that patients in the ventral-TAPP group reported significantly reduced pain, as indicated by the VAS, and better QoL within one week of surgery, as reflected in the EuraHS QoL scores for "pain at site" and "restriction of activity" domains. A study by Kelly demonstrated that the VAS score remained unaffected by variables such as age, gender, or pain's etiology [18]. This study corroborates the validity of our findings given the significant gender variation in our sample. Additionally, the length of hospital stay was significantly shorter for the ventral-TAPP group. These results align with a propensity-score matched analysis of ventral-TAPP vs. laparoscopic IPOM for small- and mid-sized ventral hernias, which reported lower postoperative pain and shorter hospital stay durations in the ventral-TAPP group compared to the IPOM group [19].

The ventral-TAPP procedure had a significantly longer operating time than the IPOM-plus procedure (P=0.004), largely due to the additional steps of creating and subsequently resuturing a peritoneal flap after mesh fixation. Despite the extended operating time, the ventral-TAPP procedure is considered cost-effective, given the higher cost of the composite or dual-sided meshes used in the IPOM-plus procedure.

The subcutaneous hematoma was identified in two patients from the ventral-TAPP group and one patient from the IPOM-plus group. The IPOM-plus group showed significantly higher seroma formation (three patients) than the ventral-TAPP group (one patient). Two patients in the ventral-TAPP group and one patient in the IPOM-plus group experienced wound infections. At the three-month follow-up, one patient from the IPOM-plus group reported intestinal obstruction, which improved with conservative management. All these findings were consistent with existing literature [20,21].

In conclusion, aside from the longer operating time, the ventral-TAPP procedure offers several advantages over the IPOM-plus repair. Our study findings suggest that the ventral-TAPP procedure is more cost-effective, associated with lower rates of seroma formation, earlier hospital discharge, lower pain scores, and better QoL to one week. These results highlight the potential benefits of the ventral-TAPP approach in terms of cost savings and improved outcomes.

Nevertheless, the IPOM-plus procedure remains a viable option, particularly in situations where there are large numbers of patients relative to available operating room time or where the surgeon may lack the technical expertise to create a peritoneal flap. This assertion is supported by the lack of significant differences in the one-month and three-month QoL scores or postoperative complications between the two procedures.

This study has several limitations. First, the small study population, being drawn from a single center with all procedures performed by a single surgical unit, limits the generalizability of our findings. Second, the short follow-up duration of three months also presents a limitation. One patient in the IPOM-plus group reported intermittent obstruction at the three-month follow-up, which could potentially occur in both groups due to inadvertent damage to the peritoneal flap, extensive foreign body reaction to the composite flap, or issues with tackers. To accurately assess the incidence of intestinal obstruction, sinus formation, enterocutaneous fistulation, and hernia recurrence, a longer follow-up period with a larger study population is needed.

Further research is also needed to assess the long-term outcomes of different LVHR techniques and their modifications. For instance, the IPOM-plus procedure required more tackers to secure the mesh compared to the ventral-TAPP procedure, where the suture-approximated peritoneum provides additional support for the mesh. This factor might contribute to the higher VAS for pain observed in the IPOM-plus group. As such, additional studies are needed to delve deeper into these aspects and improve our understanding of the relative benefits and drawbacks of these surgical procedures.

## Conclusions

Despite having longer operative time, the ventral-TAPP procedure was associated with shorter hospital stays, improved scores for "pain at the site" and "restriction of activity" in the EuraHS QoL assessment, and a more cost-effective treatment overall. Hence, our results suggest that the ventral-TAPP procedure should be the preferred choice for LVHR. However, it is important to note that in scenarios where there is a high volume of patients or where the surgeon does not possess the requisite technical expertise for performing the ventral-TAPP procedure, the IPOM-plus procedure remains a valid option. The selection between these two procedures should be individualized, taking into account the specific circumstances and resources available.
